# Knockout of AKAP150 improves impaired BK channel‐mediated vascular dysfunction through the Akt/GSK3β signalling pathway in diabetes mellitus

**DOI:** 10.1111/jcmm.15143

**Published:** 2020-03-12

**Authors:** Yan‐Rong Zhu, Xiao‐Xin Jiang, Peng Ye, Zhi‐Mei Wang, Yaguo Zheng, Zhizhong Liu, Shao‐Liang Chen, Dai‐Min Zhang

**Affiliations:** ^1^ Department of Cardiology Nanjing First Hospital Nanjing Medical University Nanjing China

**Keywords:** A‐kinase anchoring protein, diabetes mellitus, the large‐conductance Ca^2+^‐activated K^+^ channel, vascular diseases, vascular smooth muscle cells

## Abstract

Vascular dysfunction resulting from diabetes is an important factor in arteriosclerosis. Previous studies have shown that during hyperglycaemia and diabetes, AKAP150 promotes vascular tone enhancement by intensifying the remodelling of the BK channel. However, the interaction between AKAP150 and the BK channel remains open to discussion. In this study, we investigated the regulation of impaired BK channel‐mediated vascular dysfunction in diabetes mellitus. Using AKAP150 null mice (AKAP150^−/−^) and wild‐type (WT) control mice (C57BL/6J), diabetes was induced by intraperitoneal injection of streptozotocin. We found that knockout of AKAP150 reversed vascular remodelling and fibrosis in mice with diabetes and in AKAP150^−/−^ diabetic mice. Impaired Akt/GSK3β signalling contributed to decreased BK‐β_1_ expression in aortas from diabetic mice, and the silencing of AKAP150 increased Akt phosphorylation and BK‐β_1_ expression in MOVAS cells treated with HG medium. The inhibition of Akt activity caused a decrease in BK‐β_1_ expression, and treatment with AKAP150 siRNA suppressed GSK3β expression in the nuclei of MOVAS cells treated with HG. Knockout of AKAP150 reverses impaired BK channel‐mediated vascular dysfunction through the Akt/GSK3β signalling pathway in diabetes mellitus.

## 1 BACKGROUND

Type 1 diabetes mellitus is characterized by the destruction of pancreatic beta cells mediated by the immune system, resulting in reduced insulin secretion and lifelong dependence on exogenous insulin.^1^ Diabetes is an independent risk factor for vascular complications (such as atherosclerosis of the coronary artery and stroke) and a main cause of the prevalence and mortality of cardiovascular diseases.^2^, ^3^ Vascular dysfunction results from a chronic hyperglycaemic state, which leads to increased oxidative stress, vascular fibrosis and thickening, resulting in arteriosclerosis.^4^, ^5^, ^6^, ^7^ In diabetic patients, arteriosclerosis is an important and dangerous complication.

There are 4 pore‐forming subunits (BK‐α) and 4 auxiliary subunits, β subunits (BK‐β) or γ subunits (BK‐γ_1_), in the vascular large‐conductance Ca^2+^‐activated K^+^ (BK) channel. Under both physiological and pathological conditions, BK channels play a vital role in regulating vascular tension. In both type 1 and type 2 diabetic animal models, BK channel function in the vasculature is impaired, and impairment of this function is associated with decreased BK‐β_1_ expression in vascular smooth muscle cells (VSMCs).^8^, ^9^, ^10^ In diabetes mellitus, BK channel dysfunction leads to abnormal vasoconstriction and finally contributes to tissue ischaemia.

A‐kinase anchoring protein 150 (murine AKAP150, a homologue of human AKAP79) interacts with many signalling molecules, such as protein kinase A/C (PKA/PKC), calmodulin (CaM) and calmodulin‐dependent phosphatase (CaN), to regulate vascular tone and blood pressure.^11^, ^12^, ^13^ CaN is the key molecule necessary for insulin secretion in beta cells, and CaN can improve insulin sensitivity, which means CaN is also a determinant of metabolic processes. By tethering CaN, AKAP150 can regulate these processes. Thus, it can be seen that AKAP150 is a key regulatory molecule in diabetes.^14^ During hyperglycaemia and diabetes, AKAP150 promotes vascular tone enhancement by intensifying the remodelling of the BK channel. AKAP150 anchors CaN and mediates nuclear factor of activated T cells c3 (NFATc3) activation and the transcriptional suppression of regulatory BK‐β_1_ subunits under hyperglycaemic conditions.^12^ During hyperglycaemia and diabetes, enhanced vascular tone results from impaired BK channels, and the process depends on the combination between CaN and AKAP150.

Akt (protein kinase B or PKB), which is mediated through serine and/or threonine phosphorylation of downstream substrates, regulates a series of cellular processes, such as metabolism, proliferation, cell survival, growth and angiogenesis. Glucose uptake by muscle and fat cells is regulated by Akt, which relays the translocation of glucose transporter 4 to cell membranes. Akt deregulation has been implicated in diabetes.^15^ Glycogen synthase kinase‐3β (GSK3β) is a multifunctional kinase that plays crucial roles in various key biological processes, including cell proliferation, glycogen metabolism and neuronal function.^16^ GSK3β is considered a negative regulator in the insulin‐related signalling pathway, and GSK3β is inactivated when it is phosphorylated. GSK3β is a main downstream target of the Akt signalling pathway. Diabetes impairs the insulin signalling pathways and can influence cell death.^17^ GSK3β plays an important role in diabetes. The absence of the phosphorylated form of this kinase in diabetic vasculature compared with healthy vasculature is associated with increased vascular injuries. GSK3β presents as an active form and is predominantly a cytosolic protein; however, it also exists in the nucleus and mitochondria. In the nucleus and mitochondria, activation of apoptotic signalling promotes the active form of GSK3β.^18^, ^19^ During diabetes, the changed distribution of different forms of GSK3β is still unclear.

A previous study demonstrated that in streptozotocin (STZ)‐induced diabetic mouse aortas, impaired Akt phosphorylation signalling resulted in an acceleration of BK‐β_1_ protein degradation because of the overproduction of reactive oxygen species.^3^ In diabetic vasculopathy, not only is an increase in reactive oxygen species production observed but also VSMC proliferation and vascular fibrosis and thickening are observed. These data suggest that AKAP150 is critical for insulin secretion and for the relationship between BK‐β_1_ and Akt protein. However, a direct molecular mechanism between AKAP150 and BK channels has not been established during diabetes mellitus.

In this study, we employed biochemical and molecular approaches to determine whether AKAP150 associates with BK channels in diabetes and high glucose (HG) conditions. In type 1 diabetic mice, aortic vascular remodelling and fibrosis are obvious in vivo, but knockout of the AKAP150 gene can inhibit vascular dysfunction. The expression levels of the BK‐β_1_ subunit, p‐Akt473 and p‐GSK3β are suppressed in wild‐type (WT) mice but not in AKAP150‐null (AKAP150^−/−^) STZ‐induced diabetic mice. In MOVAS cells cultured in vitro with HG, using small interfering RNA (siRNA) to knock down AKAP150 can suppress cell proliferation and improve BK‐β_1_ subunit and p‐Akt473 expression. The inhibition of p‐Akt473 decreases BK‐β_1_ expression. Under HG conditions, GSK‐3β was increased in the nucleus by several folds, but the cytosolic levels were not affected in HG‐treated MOVAS cells with AKAP150 interference by siRNA, and intranuclear GSK3β expression was inhibited. The results from this study demonstrate that AKAP150 is a main ingredient of BK channel suppression through the Akt/GSK3β signalling pathway that thus contributes to vascular dysfunction during type 1 diabetes.

## 2 MATERIAL AND METHODS

### 2.1 Animals and treatment

AKAP150 null mice (AKAP150^−/−^) and WT control mice (C57BL/6J) were purchased from Jackson Laboratory at 4 weeks of age. All mice were housed at room temperature (23 ± 2°C) and 60% humidity with a 12:12‐hour light/dark cycle and unrestricted access to standard chow and water. After adaptation for 2 weeks, the mice were randomly divided into four treatment groups: the WT group (n = 30), the DM group (n = 30), the AKAP150^−/−^ group (n = 30) and the AKAP150^−/−^ DM group (n = 30). Diabetes was induced by the intraperitoneal injection of STZ (60 mg/kg/d, dissolved in 0.1 mol/L citrate buffer, pH 4.5; Sigma‐Aldrich) for 3 days. Mice with blood glucose levels ≥16.7 mmol/L 1 week after STZ injection were considered diabetic and were maintained for another 12 weeks before randomization. In addition, bodyweight and blood glucose levels were recorded weekly after STZ induction. All animal experiments were approved by the Institutional Animal Care and Use Committee of Nanjing Medical University (Nanjing, China). Experimental mice were maintained on a 12:12‐hour light‐dark cycle at 22 ± 2°C in the animal facility at Nanjing First Hospital (Jiangsu, China). All animal experiments were performed according to the NIH guidelines. At the end of each experiment, animals were anesthetized with 5% isoflurane and killed by cervical dislocation.

### 2.2 Histological analysis

Isolated mouse aortas were removed, fixed in a 4% paraformaldehyde solution and embedded in paraffin. The aortas were cut along the cross section into 5‐μm‐thick sections for haematoxylin and eosin (HE) and Sirius red staining. Images were acquired using a light microscope (original magnification, 200X; Nikon).

### 2.3 Cell culture and treatment

MOVAS, a mouse vascular smooth muscle cell line, was obtained from the Shanghai Institute of Biochemistry and Cell Biology (Shanghai, People's Republic of China). Cells were cultured in DMEM (Gibco Invitrogen) containing 25 mmol/L D‐glucose supplemented with 10% foetal bovine serum and 200 μg/mL geneticin (G418; Gibco Invitrogen) in humid air with 5% CO_2_ at 37°C. During the experiment, cells were cultured in minimal essential medium (3% FBS) under normoglycemic conditions (5.5 mmol/L glucose) for 12 hours, followed by exposure to HG (33 mmol/L glucose) conditions for various periods of time. Passages 3‐8 were used for the experiments. Akt phosphorylation was specifically inhibited by adding MK‐2206 2HCl (2.5 μmol/L; Selleck Chemicals).

### 2.4 siRNA transfection of MOVAS cells

AKAP150 siRNA (5′‐GCAAAGAGAGUCGUCAUUTTAAUGACGACUUC UCUUUGCTT‐3′) was obtained from Dharmacon. MOVAS cells were transfected with Lipofectamine RNAiMAX Reagent (Invitrogen) according to the manufacturer's protocol. A scrambled probe was used as a negative control. At 24 hours after transfection, the normal glucose‐containing medium was replaced with HG medium. At 48 hours after transfection, cells were used for further analyses.

### 2.5 Immunofluorescence staining

The expression of BK‐α and BK‐β_1_ and the colocalization of AKAP150 and BK‐β_1_ were examined by immunofluorescence. After being fixed and permeabilized, aortic tissue sections from the four groups were incubated with antibodies against AKAP150, BK‐α, BK‐β_1_ and GSK3β at a 1:200 dilution at 4°C overnight. BK‐α/AKAP150 was labelled with Alexa Fluor 488 (green) goat anti‐rabbit antibody, and BK‐β_1_/GSK3β was labelled with Alexa Fluor 555 (red) goat antimouse antibody. Then, the sections were counterstained with DAPI (blue). Finally, fluorescence images were acquired using a confocal laser scanning microscope.

### 2.6 Western blot analysis

Freshly isolated mouse aortas and cultured MOVAS cells were homogenized in RIPA lysis buffer containing protease and phosphatase inhibitors (Roche). The concentrations of soluble proteins were detected with the Pierce BCA Protein Assay (Thermo Fisher Scientific). Equal amounts of total extracted proteins (30‐60 μg) were separated by 8% or 10% sodium dodecyl sulphate‐polyacrylamide gel electrophoresis (SDS‐PAGE) and transferred to polyvinylidene difluoride (PVDF) membranes. Non‐specific protein binding was blocked with blocking buffer (5% non‐fat milk, 20 mmol/L Tris‐HCl, pH 7.6, 150 mmol/L NaCl and 0.05% Tween‐20) for 2 hours at room temperature. Next, the membranes were incubated with primary antibodies against GAPDH, β‐Actin, p‐Akt (Ser473), p‐Akt (Thr 308) and total Akt (1:1000; Cell Signaling Technology Inc); AKAP150 and BK‐α (1:500; Santa Cruz Biotechnology); and BK‐β_1_ (1:1000; Abcam Inc) in 5% BSA at 4°C overnight under continuous shaking. Then, the membranes were washed in Tris‐buffered saline with Tween‐20 (1x TBST) three times for 10 minutes each. After incubation with a secondary antibody (1:1,000; Cell Signaling Technology) for 2 hours, immunoreactive protein bands were visualized by chemiluminescence using a Syngene Bio Imaging Device (Syngene). The immunoreactive band density was analysed using ImageJ software (National Institutes of Health).

### 2.7 Statistical analysis

All continuous variables are expressed as the means ± standard errors of the mean (SEMs) and were analysed using GraphPad Prism 7.0 software. The data were compared using a paired or unpaired Student's *t* test to evaluate the statistical significance of the differences between the two groups when appropriate. Two‐way analysis of variance (ANOVA) was used to compare differences between multiple groups. Statistical significance was defined as *P* < .05.

## 3 RESULTS

### 3.1 Characterization of STZ‐induced diabetic mice

The average bodyweights of WT diabetic mice and AKAP150^−/−^ diabetic mice were 23.9 ± 0.5 g (n = 30) and 25 ± 0.8 g (n = 30), respectively. Blood glucose levels of WT diabetic mice and AKAP150^−/−^ diabetic mice were 28 ± 3 mmol/L (n = 30) and 26 ± 4 mmol/L (n = 30), respectively. To some extent, global genetic ablation of AKAP150 impacted blood glucose levels in diabetic mice compared with the corresponding WT diabetic mice. Although AKAP150^−/−^ mice globally secrete less insulin, insulin‐sensitive peripheral tissues such as skeletal muscle exhibited an improved ability to clear blood glucose due to the increased phosphorylation of Akt/PKB and activation of AMPK, which resulted in improved insulin sensitivity.^14^

### 3.2 Knockout of AKAP150 attenuates aortic vascular remodelling and fibrosis in mice with diabetes

**Figure 1:**
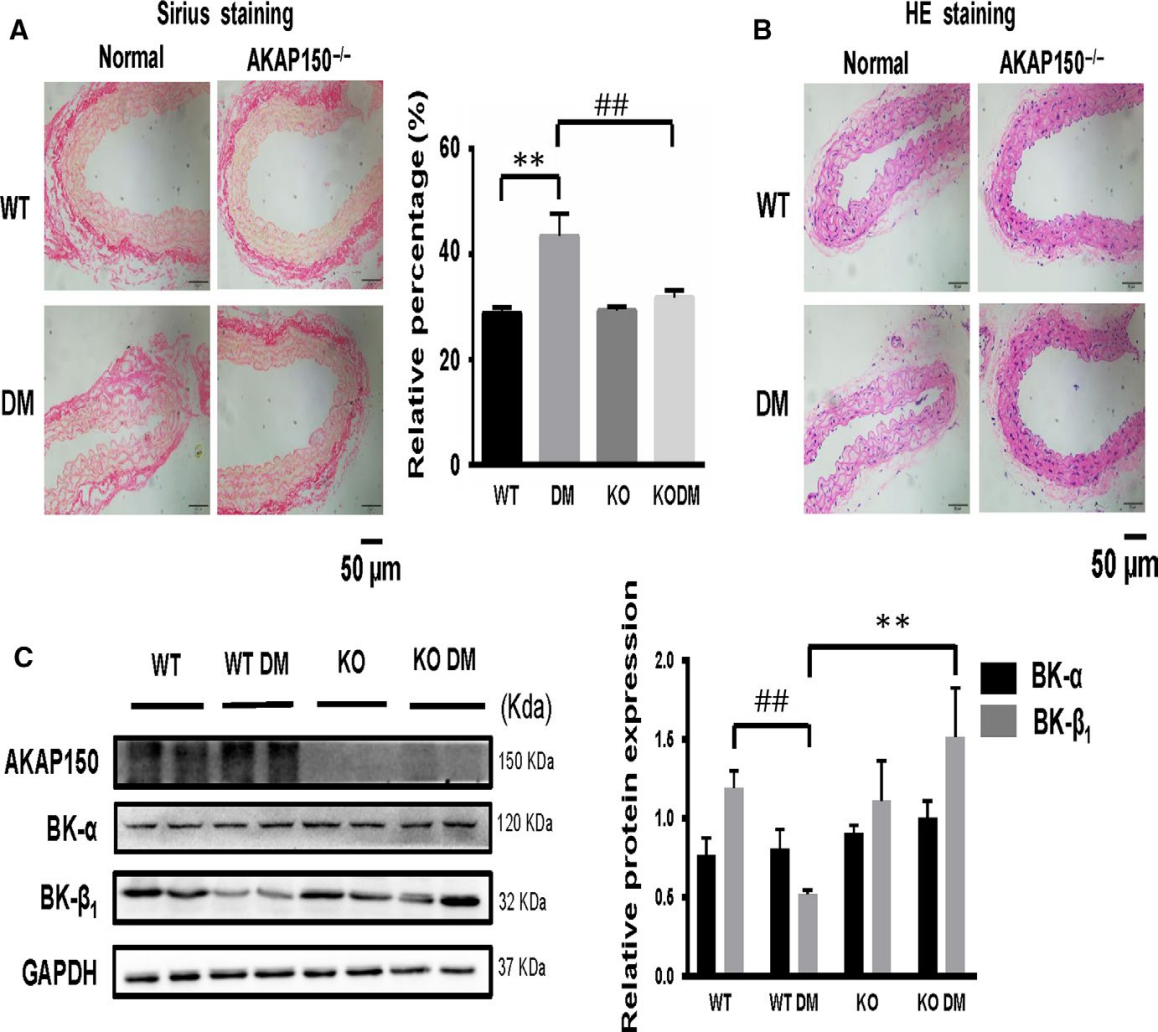
Knockout of AKAP150 reverses aortic remodelling and fibrosis by repairing the function of BK channels in mice with diabetes. A, Sirius red staining of aortas from four different groups (n = 6, each). There was no marked difference between the WT group and the AKAP150^−/−^ group. The fibrosis grade in the DM group was higher than that in the control group or AKAP150^−/−^ group. In the AKAP150^−/−^ DM group, the level of aortic fibrosis was markedly decreased compared to that in the DM group(^**^ *P * < .01 DM vs WT, n=6; ^##^ *P * < .01 DM KO vs DM, n=6). B, HE staining showed that vascular remodelling was obvious in the DM group, but in the AKAP150^−/−^ DM group, vascular remodelling was better than that in the DM group (n = 6, each). C, Western blot results revealed a decreased level of BK‐β_1_ expression in the DM group, and in the AKAP150^−/−^ DM group, BK‐β_1_ expression was higher than that in the DM group (data represent the mean ± SEM from 4 independent experiments). Two‐way ANOVA followed by Tukey's multiple comparisons test was used to determine statistically significant differences between different groups. ^##^ *P* < .01 DM vs WT, n = 4; **P < .01 KO DM vs DM, n = 4

To investigate the effect of AKAP on vascular remodelling and fibrosis, we first compared the Sirius red staining and HE staining of aortic vasculature derived from the four treatment groups: the WT group (n = 6), the DM group (n = 6), the AKAP150^−/−^ group (n = 6) and the AKAP150^−/−^ DM group (n = 6). By morphometric and histological assessments, there was no marked difference between the WT group and the AKAP150^−/−^ group. Perivascular Sirius red staining and HE staining were obvious in the DM group, which means that the remodelling and fibrosis grade of the DM group were higher than those of the WT group or AKAP150^−/−^ group (*P* < .01). In the AKAP150^−/−^ DM group, the level of aortic vascular fibrosis was markedly decreased compared to that in the DM group (Figure 1A,B). The results of the histological analysis suggested that knockout of AKAP150 attenuates aortic vascular remodelling and fibrosis in mice with diabetes.

### 3.3 BK‐β_1_ protein expression is reduced in diabetic mice but reversed in AKAP150^−/−^ DM mice

**Figure 2:**
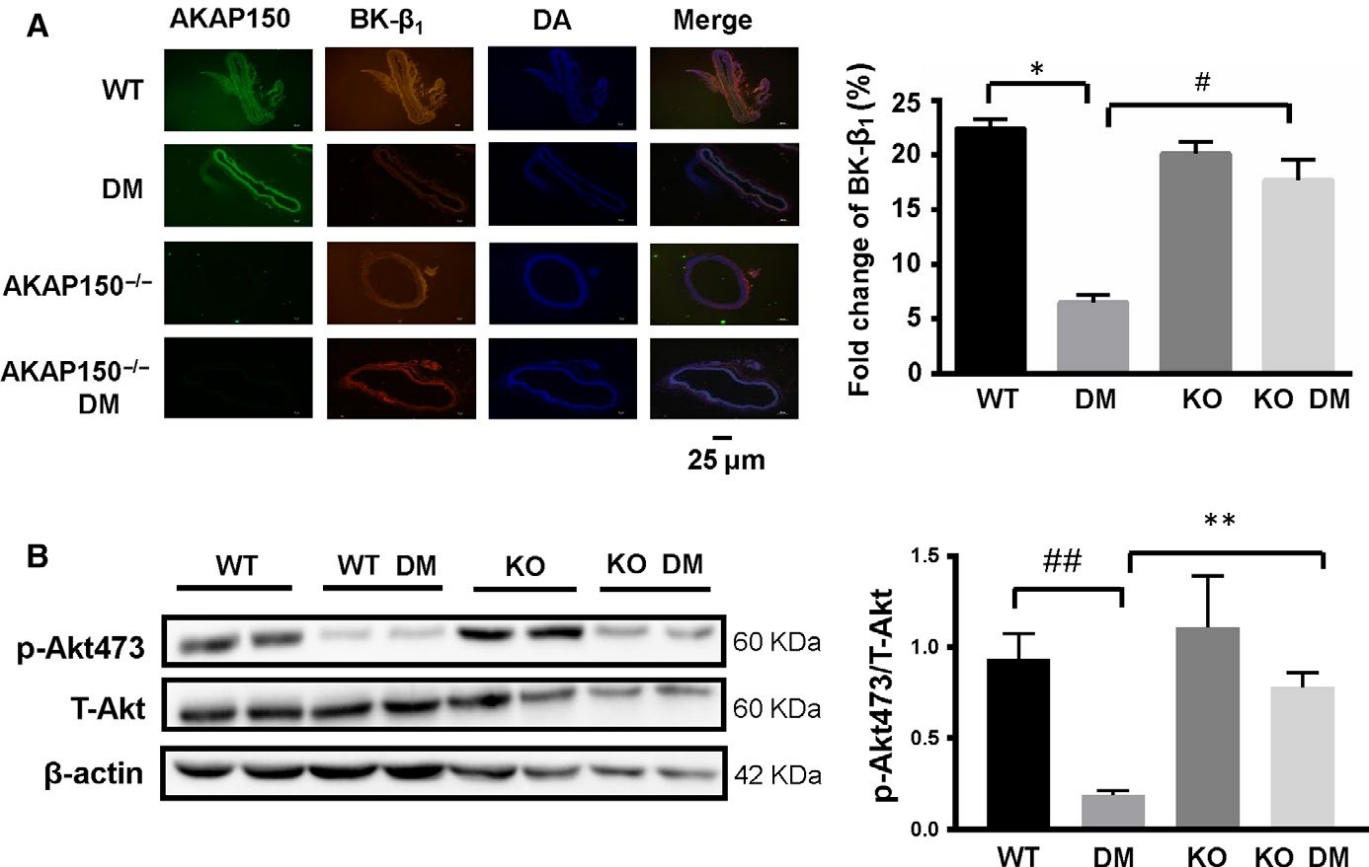
Impaired Akt signalling contributes to decreased BK‐β_1_ expression in diabetic mouse aortas. A, AKAP150 (488 nm, green), BK β_1_‐subunit (555 nm, red) and DAPI (340 nm, blue) staining suggested that BK‐β_1_ protein expression decreased in diabetic mice but increased in AKAP150^−/−^ DM mice. Knockout of AKAP150 can prevent the reduction in BK‐β_1_ protein in STZ‐induced diabetic mice(^*^ *P* < .05 DM vs WT, n=4; ^#^ *P* < .05 KO DM vs DM, n=4). B, Western blotting showed that p‐Akt473 expression decreased in diabetic mouse aortas and that p‐Akt473 expression was restored in the KO DM group (data represent the mean ± SEM from 4 independent experiments). Two‐way ANOVA followed by Tukey's multiple comparisons test was used to determine statistically significant differences between different groups. ^##^ *P* < .01 DM vs WT, n = 4; **P < .01 KO DM vs DM, n = 4

BK‐β_1_ protein levels were decreased (^##^ *P* < .01 DM vs WT, n = 4) in STZ‐induced diabetic mice (Figure 1C), whereas BK‐α expression remained unchanged. The BK‐β_1_ protein levels were significantly reversed in the AKAP150^−/−^ DM group (**P < .01 KO DM vs DM, n = 4). The same phenomenon was observed under a fluorescence microscope. Immunofluorescent staining of AKAP150, the BK α‐subunit (488 nm, green), the BK β_1_‐subunit (555 nm, red) and DAPI (340 nm, blue) in the aortic vasculature from the four groups showed that BK‐β_1_ protein expression in the DM group was lower than that in the other groups and that BK‐β_1_ protein expression increased in the AKAP150^−/−^ DM group compared to the DM group (Figure 2A). We further observed that BK‐β_1_ protein expression decreased in diabetic mice but increased in AKAP150^−/−^ DM mice. Knockout of AKAP150 can prevent the reduction of BK‐β_1_ protein expression in STZ‐induced diabetic mice.

### 3.4 Impaired Akt signalling contributes to decreased BK‐β_1_ expression in diabetic mouse aortas

We further determined the role of Akt signalling in the regulation of BK‐β_1_ expression in diabetic mouse aortas. Hyperglycaemia reduced p‐Akt473 in the aortas of diabetic mice (^##^ *P* < .01 DM vs WT, n = 4) but did not alter the level of total Akt (Figure 2B). However, p‐Akt473 protein expression was reversed in the AKAP150^−/−^ DM group (**P < .01 KO DM vs DM, n = 4). Thus, we speculated that knockout of AKAP150 leads to the up‐regulation of BK‐β_1_ subunits during diabetes through the Akt signalling pathway.

### 3.5 Silencing AKAP150 increases the levels of Akt phosphorylation and BK‐β_1_ expression in MOVAS cells treated with HG culture

**Figure 3:**
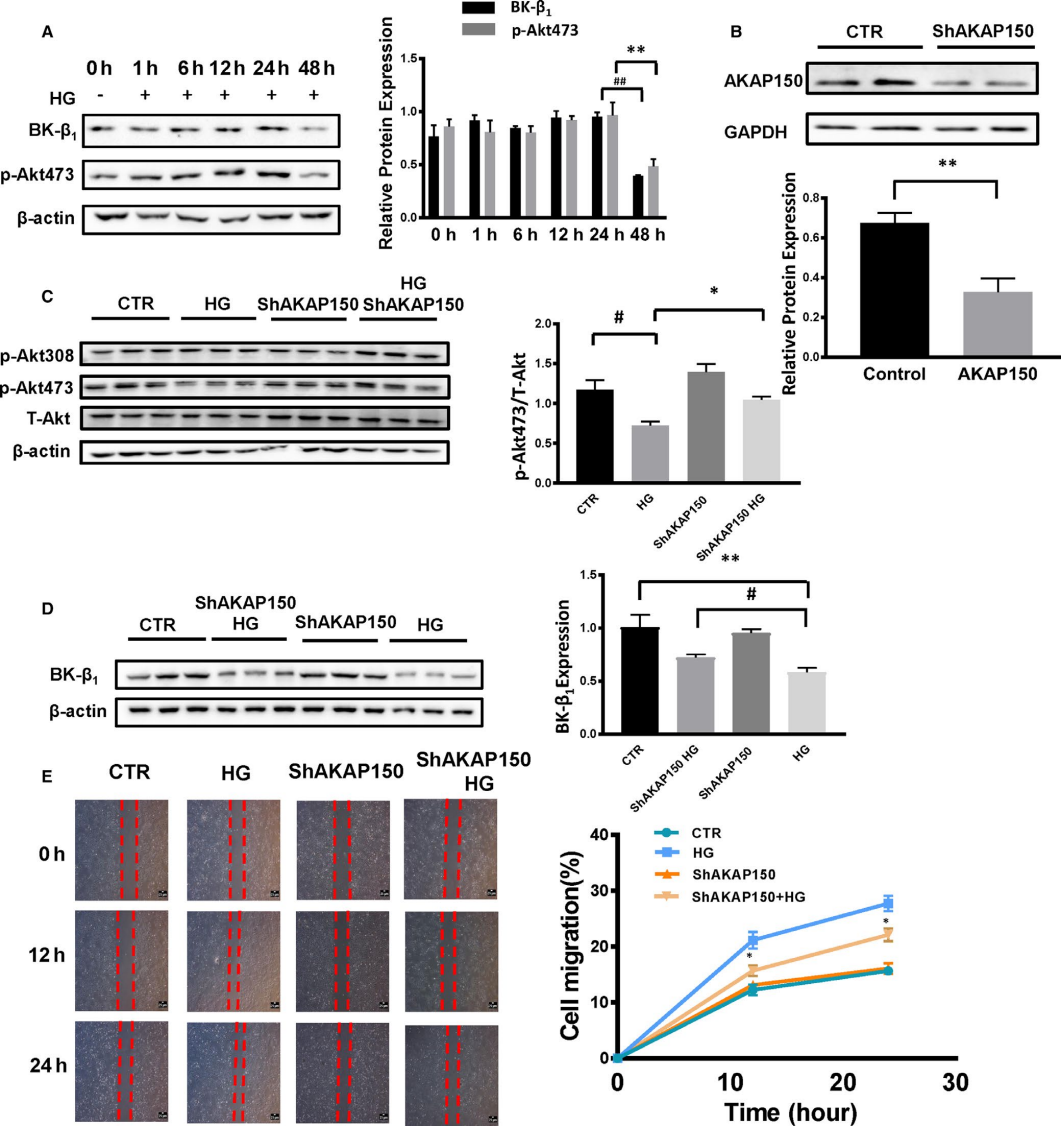
Silencing AKAP150 increases the levels of Akt phosphorylation and BK‐β_1_ expression in MOVAS cells treated with HG. A, MOVAS cells were exposed to HG for different time periods (0, 1, 6, 12, 24 and 48 h), and then, proteins were detected by Western blotting. At 48 h, BK‐β_1_ and p‐AKT473 protein expression levels were remarkably reduced (**P < .01 48 h vs 0 h, n = 3) (^##^ *P* < .01 48 h vs 0 h, n = 3). B, MOVAS cells were transfected with AKAP150 siRNA, and siRNA reduced AKAP150 expression by approximately 50% when compared to control cells (**P < .01 vs CTR, n = 3). C, Akt phosphorylation at Thr308 did not produce any differences between the four groups; however, p‐AKT473 expression decreased in MOVAS cells treated with HG (^#^ *P* < .05 HG vs CTR, n = 3). By silencing the AKAP150 gene, the levels of Akt phosphorylation in MOVAS cells treated with HG obviously increased (*P < .05 siAKAP150 HG vs HG, n = 3). D, BK‐β_1_ expression followed the same trend as p‐Akt473 and increased in the siAKAP150 HG group compared to the HG group (^#^ *P* < .05 siAKAP150 HG vs HG, n = 3) (**P < .01 HG vs CTR, n = 3). E, Cell migration assessed by the wound healing test showed that the proliferative and migrative effects of HG were inhibited by AKAP150 siRNA (*P < .05 vs HG, n = 3). All results represent the mean ± SEM from 3 independent experiments. Two‐way ANOVA followed by Tukey's multiple comparisons test was used to determine statistically significant differences between different groups. *P < .05; ^#^ *P* < .05; **P < .01; ^##^ *P* < .01

Considering the regulation of vascular BK‐β_1_ expression in cultured smooth muscle cells by Akt signalling, the role of AKAP150 in BK channel‐mediated artery dysfunction in diabetes is critical. Confirming the effects of HG culture on BK‐β_1_ expression and Akt phosphorylation, we found reduced BK‐β_1_ expression and Akt phosphorylation at Ser473 in a time‐dependent manner. p‐Akt473 reached a steady‐state level after 48 hours (**P < .01 48 hours vs 0 hour, n = 3) (^##^ *P* < .01 48 hours vs 0 hour, n = 3) (Figure 3A). The role of AKAP150 HG‐induced phosphorylation of Akt was studied using siRNA, and siRNA reduced AKAP150 expression by approximately 50% when compared to that of the control cells (**P < .01 vs CTR, n = 3) (Figure 3B). No differences in Akt phosphorylation at Thr308 were observed between the four groups; however, p‐Akt473 expression decreased in MOVAS cells treated with HG (^#^ *P* < .05 HG vs CTR, n = 3). By silencing the AKAP150 gene, the levels of Akt phosphorylation in MOVAS cells treated with HG obviously increased (*P < .05 siAKAP150 HG vs HG, n = 3) (Figure 3C). BK‐β_1_ expression showed similar behaviour to p‐Akt473 and increased in the siAKAP150 HG group compared to the HG group (^#^ *P* < .05 vs HG, n = 3) (Figure 3D).

### 3.6 A proliferative role for AKAP150 in cells treated with HG

AKAP150 plays an important role in MOVAS proliferation and migration. We showed that the proliferative and migrative effects of HG were inhibited by AKAP150 siRNA. Cell migration was assessed by the wound healing test (Figure 3E). The migration of cells treated with HG for 12 and 24 hours was higher than that of cells treated with AKAP150 siRNA HG (*P < .05 vs HG, n = 3). These data indicated a proliferative role for AKAP150 in the aortas of type 1 diabetic mice.

### 3.7 The inhibition of Akt activity caused a decrease in BK‐β_1_ expression

**Figure 4:**
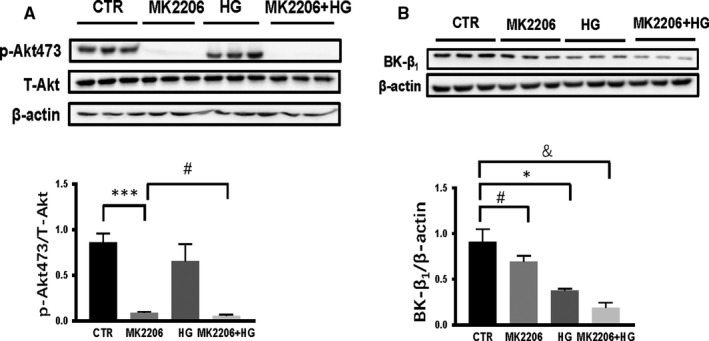
The inhibition of Akt activity caused decreased BK‐β_1_ expression. A, p‐Akt expression in MOVAS cells treated with MK‐2206, a selective blocker of Akt phosphorylation, markedly decreased by 90% (***P < .001 MK‐2206 vs CTR, n = 3) (^###^ *P* < .001 vs CTR, n = 3). B, In MK‐2206‐treated cells, BK‐β_1_ expression decreased and was the same as that in the HG‐treated group (**P < .01 vs CTR, n = 3) (^##^ *P* < .01 vs CTR, n = 3). In the CTR, MK‐2206, HG and MK‐2206 HG groups, BK‐β_1_ expression exhibited a gradual downward trend (^#^ *P* < .05 vs CTR, n = 3) (*P < .05 vs CTR, n = 3) (^&^ *P* < .05 vs CTR, n = 3). All results represent the mean ± SEM from 3 independent experiments. Two‐way ANOVA followed by Tukey's multiple comparisons test was used to determine statistically significant differences between different groups. *P < .05; ^#^ *P* < .05; ^&^ *P* < .05; **P < .01; ^##^ *P* < .01; ***P < .001; ^###^ *P* < .001

Next, we examined whether the inhibition of Akt activity contributes to variations in BK‐β_1_ expression. We used MK‐2206 (a selective blocker of Akt phosphorylation) to inhibit Akt activity. As shown in Figure 4A, p‐Akt expression in MOVAS treated with MK‐2206 markedly decreased by 90% (***P < .001 MK‐2206 vs CTR, n = 3) (^###^ *P* < .001 vs CTR, n = 3). BK‐β_1_ expression decreased. In the CTR, MK‐2206/HG and MK‐2206 HG groups, BK‐β_1_ expression exhibited a gradual upward trend (^#^ *P* < .05 vs CTR, n = 3) (*P < .05 vs CTR, n = 3) (^&^ *P* < .05 vs CTR, n = 3) (Figure 4B). All of these data show that the inhibition of Akt activity contributed to a decrease in BK‐β_1_ expression. AKAP150‐regulated BK channels during diabetes depend on the Akt signalling pathway.

### 3.8 Knockout of AKAP150 increased the level of GSK3β phosphorylation in diabetic mice and attenuated the active form of GSK3β in MOVAS cells treated with HG

**Figure 5:**
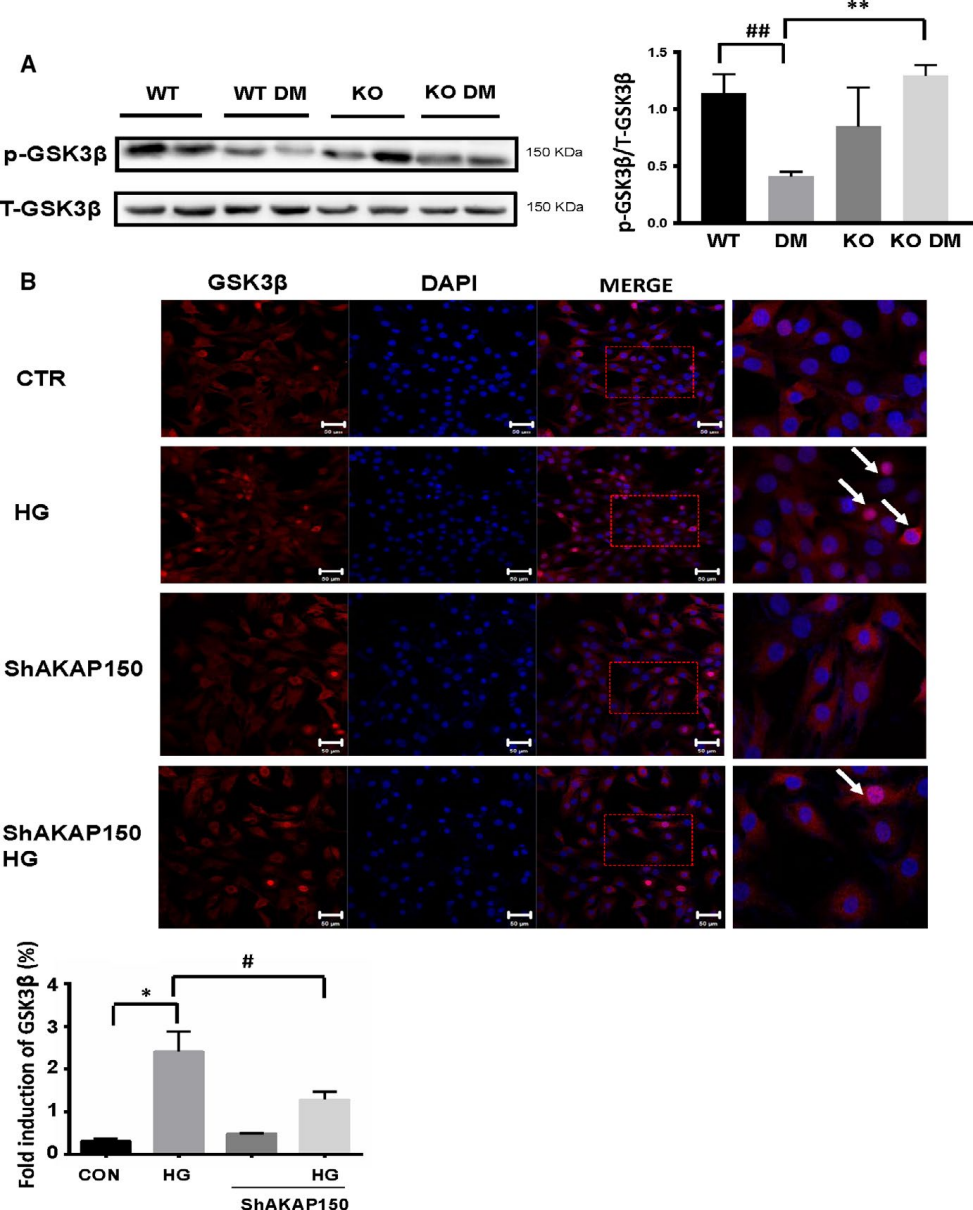
Knockout of AKAP150 increased GSK3β phosphorylation levels in diabetic mice and suppressed GSK3β expression in the nuclei of MOVAS cells treated with HG. A, Western blotting showed aortic proteins from four different treatment groups. p‐GSK3β expression showed an obvious decrease in the DM group, similar to the decrease observed for p‐Akt473 expression (^##^ *P* < .01 DM vs WT, n = 4). In the KO DM group, the p‐GSK3β protein level increased compared to that in the DM group (**P < .01 KO DM vs DM, n = 4). B, Immunofluorescence staining was used to detect GSK3β nuclear translocation. Under normal conditions, GSK3β was predominantly a cytosolic protein, and a small portion may also have been present in the nuclei. Under HG conditions, GSK3β levels increased in the nucleus by several folds, but HG conditions did not affect cytosolic levels. In HG‐treated MOVAS cells with AKAP150 siRNA, intranuclear GSK3β expression was inhibited(^*^ *P* < .05 HG vs CON, n=4; ^#^ *P* < .05 HG vs ShAKAP150 HG, n=4). All results represent the mean ± SEM from 4 independent experiments. Two‐way ANOVA followed by Tukey's multiple comparisons test was used to determine statistically significant differences between different groups

To further determine the mechanism downstream of the Akt protein, we observed GSK3β, one of the isoforms of GSK3 that is associated with glycometabolism. p‐GSK3β protein levels were obviously decreased in the DM group, similar to p‐Akt473 expression (^##^ *P* < .01 DM vs WT, n = 4). However, in the KO DM group compared to the DM group, the p‐GSK3β protein level increased (**P < .01 KO DM vs DM, n = 4) (Figure 5A). We then detected GSK3β nuclear translocation. Under normal conditions, GSK3β was predominantly a cytosolic protein, and a small portion may also have been present in nuclei. Under HG conditions, GSK3β levels in the nucleus increased by several folds, but HG conditions did not affect the cytosolic levels. In HG‐treated MOVAS cells with AKAP150 interference by siRNA, intranuclear GSK3β expression was inhibited, as shown in Figure 5B. All of these results showed that in diabetic mice, GSK3β phosphorylation levels decreased, which led to the inactivation of GSK3β, and this inactivation may be related to the abnormal localization of GSK3β. Knockout of AKAP150 increased GSK3β phosphorylation levels and suppressed GSK3β expression in the nuclei of diabetic mice.

## 4 DISCUSSION

In this study, we made several novel findings. In vivo experiments revealed that (1) in aortas from type 1 diabetic mice but not AKAP150^−/−^ diabetic mice, vascular remodelling and fibrosis are obvious and that (2) BK‐β_1_ subunit, p‐Akt473 and p‐GSK3β expressions are suppressed in WT but not AKAP150^−/−^ STZ‐induced diabetic mice. In vitro experiments revealed that (1) in MOVAS cells cultured in HG medium, AKAP150 siRNA suppressed cell proliferation and increased BK‐β_1_ subunit and p‐Akt473 expression; (2) the inhibition of p‐Akt473 decreased BK‐β_1_ expression; and (3) in MOVAS cells treated with HG, the localization of GSK3β changed compared to that in MOVAS cells under normal conditions in which intranuclear GSK‐3β expression increased. Hence, our results suggest that during type 1 diabetes, AKAP150 is an important component of BK channel suppression through the Akt/GSK3β signalling pathway that contributes to vascular dysfunction.

BK channels are widely distributed in various tissues, especially in arterial vessels. BK channels are composed of four pore‐forming α subunits and four auxiliary β/γ subunits. BK‐β_1_, which is encoded by the KCNMB1 gene, influences many molecular pathways in diabetic vasculopathy. In both type 1 and type 2 diabetic animal models, BK‐β_1_ expression in VSMCs is decreased, while BK‐α subunit expression is unchanged in most animal models of type 1 DM. Adenoviral expression of the KCNMB1 gene in coronary arteries from type 1 DM mice greatly improved BK channel function.^3^, ^7^, ^8^, ^9^, ^10^, ^20^ We demonstrated that the reduction in BK‐β_1_ is truly an important factor in vascular dysfunction (Figure 2A). In VSMCs, endothelial cells and macrophages, the Akt‐included signalling network plays a major functional role in the cells that are dysregulated under abnormal conditions. Akt1 is the representative isoform expressed in VSMCs that contributes to VSMC proliferation and migration.^21^, ^22^ The insulin response also requires Akt‐related signalling. The activation of Akt is related to proper glucose uptake and the production of insulin from pancreatic cells.^22^, ^23^ Some evidence suggests that Akt phosphorylation and ubiquitination contribute to the regulation of metabolic homeostasis through the regulation of insulin signalling.^22^, ^23^, ^24^, ^25^, ^26^ Impaired Akt signalling influenced the clearance of circulating glucose. A previous study showed that in STZ‐induced diabetic mouse vessels and in HG‐cultured human coronary SMCs, down‐regulated BK‐β_1_ expression was related to impaired PI3K/Akt signalling.^3^ This effect was observed not only in vessels but also in conditional BK mutants with cardiomyocyte‐specific knockout of the BK channel (CMBK‐KO). After ischaemia and reperfusion (I/R) with 10‐min reperfusion, the p‐Akt/Akt ratio showed a significant decrease in CMBK‐KO mouse heart lysates compared with control mouse (CMBK‐CTR) heart lysates.^17^ It is obvious that the interaction between BK‐β_1_ and Akt in both diabetic models and I/R models is substantial. In this study, we further demonstrated that in type 1 diabetes or in MOVAS cells cultured in HG DMEM, the levels of both BK‐β_1_ and p‐Akt473 appeared to be concurrently reduced after 48 hours (Figures 2B and 3A). Located downstream of Akt, the forkhead box O (FoxO) subfamily is associated with BK‐β_1_. In diabetic mice, vascular BK‐β_1_ expression decreased along with the up‐regulation of transcription factor‐3a (FoxO‐3a)–dependent F‐box–only protein (FBXO).^3^, ^10^ Other proteins downstream of Akt, such as GSK3 and GSK3β, participate in various key biological processes.^27^ In our study, we found that abnormal localization of GSK3β and impaired activity of GSK3β phosphorylation may be related to dysfunction of the BK channel (Figure 5A,B).

AKAPs are distinguished by their ability to bind cyclic adenosine monophosphate (cAMP)‐PKA at focal points within the cell to ensure the integration and processing of multiple signalling pathways.^28^ AKAP150 is an AKAP5 gene product that was first identified as a scaffolding protein predominantly expressed in the cerebral cortex that anchored the RII regulatory subunit of PKA to post‐synaptic densities.^29^, ^30^, ^31^ However, abundant data have shown that AKAP150 is important in the nervous system in functions such as neuron excitability^32^ and neuroendocrine function.^33^ Additionally, AKAP150 is functionally coupled to G protein–coupled receptors (GPCRs), adenylyl cyclases (ACs) and ion channels.^12^, ^34^, ^35^ In the cardiovascular system, AKAP150 can regulate Ca^2+^ cycling, myocyte contractility and susceptibility to heart failure after pathological stress, suggesting that the AKAP150 signalling pathway may serve as a therapeutic target for heart failure.^36^

During hyperglycaemia and diabetes, AKAP150 knockdown inhibits HG‐induced apoptosis in neonatal rat cardiomyocytes.^37^ In vessels, glucose‐mediated increases in CaV1.2 channel activity were related to PKA activity, leading to α_1C_ phosphorylation at Ser1928. This is an AKAP150‐dependent, PKA‐mediated phosphorylation event. Without AKAP150, mouse arteries exhibited vasoconstriction upon acute increases in extracellular D‐glucose and in diabetes.^38^ CaN anchoring by AKAP150 is required for BK channel impairment during hyperglycaemia and diabetes, which promotes enhanced vascular tone.^12^ In our study, the function of AKAP150 in the vasculature from STZ‐induced diabetes was undeniable (Figures 1A‐C and 2A,B). AKAP150 is a modulator of glucose homeostasis. In AKAP150^−/−^ mice, less insulin was secreted from β‐cells, but the mice displayed improved glucose handling due to increased insulin sensitivity in target tissues. We also observed the same result in our experiments. In AKAP150^−/−^ mice, the absence of a seven amino acid sequence responsible for tethering CaN contributed to retained metabolically advantageous characteristics, ultimately maintaining glucose homeostasis.^39^

**Figure 6:**
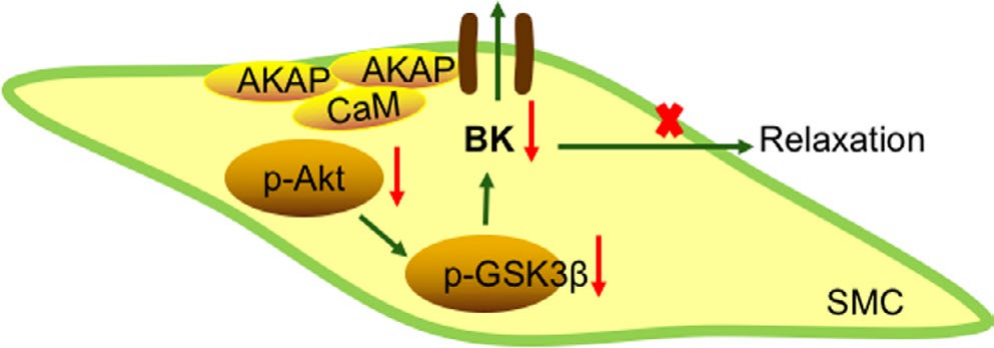
Model of regulation of BK channel activity by AKAP150 in diabetes. In diabetes, AKAP150 binds to Akt directly or indirectly, resulting in decreased phosphorylation levels of Akt at the Ser473 site. GSK3β is downstream of Akt. p‐GSK3β, which is the same as p‐Akt473, decreases. In addition, the GSK3β level increases in the nucleus, which damages the activity of the BK channel

A novel finding of our study was that in AKAP150^−/−^ DM mice compared to WT DM mice, aortic remodelling and fibrosis were inhibited by the up‐regulation of BK‐β_1_ expression, Akt phosphorylation at Ser473 and the expression of p‐GSK3β (Figures 2C and5A). In AKAP150 knockdown MOVAS cells cultured in HG medium, cell proliferation was suppressed (Figure 3E). When p‐Akt473 activity was inhibited, BK‐β_1_ was down‐regulated (Figure 4B). Hence, during hyperglycaemia and diabetes, AKAP150 binds to Akt directly or indirectly, resulting in decreased phosphorylation levels of Akt at the Ser473 site. As downstream of Akt, the GSK3β level increases in the nucleus, which damages the activity of the BK channel (Figure 6). The knockdown of AKAP150 improved BK channel‐mediated vascular dysfunction, and this protection mechanism was based on increased p‐Akt473 and p‐GSK3β activity. AKAP150 can serve as a therapeutic target for BK channel‐mediated vascular dysfunction.

## 5 CONCLUSIONS

In summary, our study indicated that knockout of AKAP150 improves impaired BK channel‐mediated vascular dysfunction through the Akt/GSK3β signalling pathway in diabetes mellitus.

## CONFLICT OF INTEREST

The authors have no conflict of interest to declare.

## AUTHOR CONTRIBUTIONS

Zhang DM conceived the project and designed research. Zhu YR, Jiang XX and Ye P performed the experiments. Wang ZM and Zhen YG performed tissue processing and assistance. Liu ZZ and Chen SL critical read the manuscript. Zhu YR and Zhang DM analysed the data and wrote the original draft. Zhang DM contributed to final editing of the manuscript. All authors read and approved the final manuscript.

## Data Availability

All data generated during the current study are available from the corresponding author on reasonable request.

## References

[1] Bjarnegard N , Arnqvist HJ , Lindstrom T , Jonasson L , Jönsson A , Länne T . Long‐term hyperglycaemia impairs vascular smooth muscle cell function in women with type 1 diabetes mellitus. Diab Vasc Dis Res. 2009;6(1):25‐31. 19156625 10.3132/dvdr.2009.005

[2] Dafoulas GE , Toulis KA , Mccorry D , et al. Type 1 diabetes mellitus and risk of incident epilepsy: a population‐based, open‐ cohort study. Diabetologia. 2017;60(2):258‐261. 27796422 10.1007/s00125-016-4142-x PMC6518067

[3] Lu T , Chai Q , Yu L , et al. Reactive oxygen species signaling facilitates FOXO‐3a/FBXO‐dependent vascular BK channel beta1 subunit degradation in diabetic mice. Diabetes. 2012;61(7):1860‐1868. 22586590 10.2337/db11-1658 PMC3379647

[4] Peyton KJ , Ensenat D , Azam MA , et al. Arginase promotes neointima formation in rat injured carotid arteries. Arterioscler Thromb Vasc Biol. 2009;29(4):488‐494. 19164802 10.1161/ATVBAHA.108.183392 PMC2662760

[5] Llaurado G , Ceperuelo‐Mallafre V , Vilardell C , et al. Arterial stiffness is increased in patients with type 1 diabetes without cardiovascular disease: a potential role of low‐grade inflammation. Diabetes Care. 2012;35(5):1083‐1089. 22357186 10.2337/dc11-1475 PMC3329819

[6] Bhatta A , Yao L , Xu Z , et al. Obesity‐induced vascular dysfunction and arterial stiffening requires endothelial cell arginase 1. Cardiovasc Res. 2017;113(13):1664‐1676. 29048462 10.1093/cvr/cvx164 PMC6410953

[7] Zhu Y , Ye P , Chen SL , Zhang DM . Functional regulation of large conductance Ca(2+)‐activated K(+) channels in vascular diseases. Metabolism. 2018;83:75‐80. 29373813 10.1016/j.metabol.2018.01.008

[8] Yi F , Wang H , Chai Q , et al. Regulation of large conductance Ca2+‐activated K+ (BK) channel beta1 subunit expression by muscle RING finger protein 1 in diabetic vessels. J Biol Chem. 2014;289(15):10853‐10864. 24570002 10.1074/jbc.M113.520940 PMC4036198

[9] Lu T , Sun X , Li Y , Chai Q , Wang XL , Lee HC . Role of Nrf2 signaling in the regulation of vascular BK channel beta1 subunit expression and BK channel function in high‐fat diet‐induced diabetic mice. Diabetes. 2017;66(10):2681‐2690. 28465407 10.2337/db17-0181 PMC5606315

[10] Zhang DM , He T , Katusic ZS , Lee HC , Lu T . Muscle‐specific F‐box only proteins facilitate BK channel 1 subunit downregulation in vascular smooth muscle cells of diabetes mellitus. Circ Res. 2010;107(12):1454‐1459. 20966391 10.1161/CIRCRESAHA.110.228361 PMC3076051

[11] Li X , Matta SM , Sullivan RD , Bahouth SW . Carvedilol reverses cardiac insufficiency in AKAP5 knockout mice by normalizing the activities of calcineurin and CaMKII. Cardiovasc Res. 2014;104(2):270‐279. 25225170 10.1093/cvr/cvu209 PMC4296113

[12] Nystoriak MA , Nieves‐Cintron M , Nygren PJ , et al. AKAP150 contributes to enhanced vascular tone by facilitating large‐conductance Ca2+‐activated K+ channel remodeling in hyperglycemia and diabetes mellitus. Circ Res. 2014;114(4):607‐615. 24323672 10.1161/CIRCRESAHA.114.302168 PMC3954117

[13] Nieves‐Cintrón M , Hirenallur‐Shanthappa D , Nygren PJ , et al. AKAP150 participates in calcineurin/NFAT activation during the down‐regulation of voltage‐gated K+ currents in ventricular myocytes following myocardial infarction. Cell Signal. 2016;28(7):733‐740. 26724383 10.1016/j.cellsig.2015.12.015 PMC4902329

[14] Teo AKK , Kulkarni RN . Setting sail for glucose homeostasis with the AKAP150‐PP2B‐anchor. EMBO J. 2012;31(20):3956‐3957. 22983555 10.1038/emboj.2012.265 PMC3474931

[15] Hers I , Vincent EE , Tavare JM . Akt signalling in health and disease. Cell Signal. 2011;23(10):1515‐1527. 21620960 10.1016/j.cellsig.2011.05.004

[16] Pandey MK , DeGrado TR . Glycogen Synthase Kinase‐3 (GSK‐3)‐targeted therapy and imaging. Theranostics. 2016;6(4):571‐593. 26941849 10.7150/thno.14334 PMC4775866

[17] Mokhtari B , Badalzadeh R , Alihemmati A , Mohammadi M . Phosphorylation of GSK‐3beta and reduction of apoptosis as targets of troxerutin effect on reperfusion injury of diabetic myocardium. Eur J Pharmacol. 2015;765:316‐321. 26341011 10.1016/j.ejphar.2015.08.056

[18] Bijur GN , Jope RS . Glycogen synthase kinase‐3 beta is highly activated in nuclei and mitochondria. Neuroreport. 2003;14:2415‐2419. 14663202 10.1097/00001756-200312190-00025

[19] Bijur GN , Jope RS . Proapoptotic stimuli induce nuclear accumulation of glycogen synthase kinase‐3 beta. J Biol Chem. 2001;276:37436‐37442. 11495916 10.1074/jbc.M105725200 PMC1973163

[20] Vetri F , Xu H , Paisansathan C , Pelligrino DA . Impairment of neurovascular coupling in type 1 diabetes mellitus in rats is linked to PKC modulation of BK(Ca) and Kir channels. Am J Physiol Heart Circ Physiol. 2012;302(6):H1274‐1274. 22268114 10.1152/ajpheart.01067.2011 PMC3311480

[21] Frankenreiter S , Bednarczyk P , Kniess A , et al. cGMP‐elevating compounds and ischemic conditioning provide cardioprotection against ischemia and reperfusion injury via cardiomyocyte‐specific BK channels. Circulation. 2017;136(24):2337‐2355. 29051185 10.1161/CIRCULATIONAHA.117.028723

[22] Ferna´ndez‐Hernando C , Jo´zsef L , Jenkins D , Di Lorenzo A , Sessa WC . Absence of Akt1 reduces vascular smooth muscle cell migration and survival and induces features of plaque vulnerability and cardiac dysfunction during atherosclerosis. Arterioscler Thromb Vasc Biol. 2009;29(12):2033‐2040. 19762778 10.1161/ATVBAHA.109.196394 PMC2796372

[23] Manning BD , Toker A . AKT/PKB Signaling: navigating the network. Cell. 2017;169(3):381‐405. 28431241 10.1016/j.cell.2017.04.001 PMC5546324

[24] Buzzi F , Xu L , Zuellig RA , et al. Differential effects of protein kinase B/Akt isoforms on glucose homeostasis and islet mass. Mol Cell Biol. 2010;30(3):601‐612. 19933838 10.1128/MCB.00719-09 PMC2812230

[25] Cederquist CT , Lentucci C , Martinez‐Calejman C , et al. Systemic insulin sensitivity is regulated by GPS2 inhibition of AKT ubiquitination and activation in adipose tissue. Mol Metab. 2017;6(1):125‐137. 28123943 10.1016/j.molmet.2016.10.007 PMC5220281

[26] Yang WL , Wang J , Chan CH , et al. The E3 ligase TRAF6 regulates Akt ubiquitination and activation. Science. 2009;325(5944):1134‐1138. 19713527 10.1126/science.1175065 PMC3008763

[27] Gong Y , Ma Y , Sinyuk M , et al. Insulin‐mediated signaling promotes proliferation and survival of glioblastoma through Akt activation. Neuro Oncol. 2016;18(1):48‐57. 26136493 10.1093/neuonc/nov096 PMC4677408

[28] Welch EJ , Jones BW , Scott JD . Networking with AKAPs: context‐ dependent regulation of anchored enzymes. Mol Interv. 2010;10(2):86‐97. 20368369 10.1124/mi.10.2.6 PMC2895371

[29] Ercu M , Klussmann E . Roles of A‐kinase anchoring proteins and phosphodiesterases in the cardiovascular system. J Cardiovasc Dev Dis. 2018;5(1):14. 10.3390/jcdd5010014 PMC5872362 29461511

[30] Skroblin P , Grossmann S , Schafer G , Rosenthal W , Klussmann E . Mechanisms of protein kinase A anchoring. Int Rev Cell Mol Biol. 2010;283:235‐330. 20801421 10.1016/S1937-6448(10)83005-9

[31] Redden JM , Dodge‐Kafka KL . AKAP phosphatase complexes in the heart. J Cardiovasc Pharmacol. 2011;58(4):354‐362. 21562429 10.1097/FJC.0b013e31821e5649 PMC3158305

[32] Jeske NA , Por ED , Belugin S , et al. A‐kinase anchoring protein 150 mediates transient receptor potential family V type 1 sensitivity to phosphatidylinositol‐4,5‐bisphosphate. J Neurosci. 2011;31(23):8681‐8688. 21653872 10.1523/JNEUROSCI.0020-11.2011 PMC3125677

[33] Chen Q , Weiner RI , Blackman BE . Decreased expression of A‐kinase anchoring protein 150 in GT1 neurons decreases neuron excitability and frequency of intrinsic gonadotropin‐releasing hormone pulses. Endocrinology. 2010;151(1):281‐290. 19887564 10.1210/en.2009-0894 PMC2803148

[34] Gao T , Yatani A , Dell’Acqua ML , et al. cAMP‐dependent regulation of cardiac L‐type Ca2+ channels requires membrane targeting of PKA and phosphorylation of channel subunits. Neuron. 1997;19(1):185‐196. 9247274 10.1016/s0896-6273(00)80358-x

[35] Fraser ID , Cong M , Kim J , et al. Assembly of an A kinase‐anchoring protein‐beta(2)‐adrenergic receptor complex facilitates receptor phosphorylation and signaling. Curr Biol. 2000;10(7):409‐412. 10753752 10.1016/s0960-9822(00)00419-x

[36] Li L , Li J , Drum BM , et al. Loss of AKAP150 promotes pathological remodelling and heart failure propensity by disrupting calcium cycling and contractile reserve. Cardiovasc Res. 2016;113(2):147‐159. 27856611 10.1093/cvr/cvw221 PMC5340143

[37] Zeng C , Wang J , Li N , et al. AKAP150 mobilizes cPKC‐dependent cardiac glucotoxicity. Am J of Physiol Endocrinol Metab. 2014;307(4):E384‐E397. 25005497 10.1152/ajpendo.00175.2014

[38] Nystoriak MA , Nieves‐Cintrón M , Patriarchi T , et al. Ser 1928 phosphorylation by PKA stimulates the L‐type Ca 2+ channel Ca V 1.2 and vasoconstriction during acute hyperglycemia and diabetes. Sci Signal. 2017;10(463):eaaf9647. 28119464 10.1126/scisignal.aaf9647 PMC5297430

[39] Hinke SA , Navedo MF , Ulman A , et al. Anchored phosphatases modulate glucose homeostasis. EMBO J. 2012;31(20):3991‐4004. 22940692 10.1038/emboj.2012.244 PMC3474922

